# Oncolytic Virus-Mediated Reversal of Impaired Tumor Antigen Presentation

**DOI:** 10.3389/fonc.2014.00077

**Published:** 2014-04-10

**Authors:** Shashi A. Gujar, Patrick W. K. Lee

**Affiliations:** ^1^Department of Microbiology and Immunology, Dalhousie University, Halifax, NS, Canada; ^2^Strategy and Organizational Performance, IWK Health Centre, Halifax, NS, Canada; ^3^Department of Pathology, Dalhousie University, Halifax, NS, Canada

**Keywords:** reovirus, oncolytic virus, immunotherapy, antigen presentation, anti-tumor immunity

## Abstract

Anti-tumor immunity can eliminate existing cancer cells and also maintain a constant surveillance against possible relapse. Such an antigen-specific adaptive response begins when tumor-specific T cells become activated. T-cell activation requires two signals on antigen presenting cells (APCs): antigen presentation through major histocombatibility complex (MHC) molecules and co-stimulation. In the absence of one or both these signals, T cells remain inactivated or can even become tolerized. Cancer cells and their associated microenvironment strategically hinder the processing and presentation of tumor antigens and consequently prevent the development of anti-tumor immunity. Many studies, however, demonstrate that interventions that over-turn tumor-associated immune evasion mechanisms can establish anti-tumor immune responses of therapeutic potential. One such intervention is oncolytic virus (OV)-based anti-cancer therapy. Here, we discuss how OV-induced immunological events override tumor-associated antigen presentation impairment and promote appropriate T cell–APC interaction. Detailed understanding of this phenomenon is pivotal for devising the strategies that will enhance the efficacy of OV-based anti-cancer therapy by complementing its inherent oncolytic activities with desired anti-tumor immune responses.

## Introduction

Anti-tumor immune response of appropriate magnitude and specificity has become a valid indicator of good prognosis of cancer and associated disease pathology (1–6). As such, many therapeutic options are being investigated for their capacity to promote anti-tumor immune responses. These immunotherapies, which are based on exploiting the functions of immune cells [e.g., T cells, dendritic cells (DCs)] or immune mediators (e.g., antibodies, cytokines), represent a highly promising group of interventions and have the potential to target a multitude of cancers. Considering the fact that the presence of tumor-specific CD8 T-cell responses almost always correlate with positive patient outcomes (3), the ultimate goal of most of these immunotherapies primarily focuses on establishing anti-tumor T-cell immunity (3, 4, 7). Fully functional tumor-specific T cells can not only eliminate existing cancer cells but also establish an active, ongoing, and long-term surveillance against possibly relapsing cancer cells. Indeed, the immunotherapy-promoted anti-tumor T-cell responses have shown to delay the onset of pathology, reduce the severity of disease, and prolong the survival of cancer-bearing hosts in animal experiments and in clinical settings (1–7).

Oncolytic viruses (OVs), in their naturally unmodified or genetically engineered form, preferentially infect and lyse transformed or cancerous cells in a process called oncolysis. Some of the more prominent examples of these OVs include adenoviruses, reovirus, herpes simplex virus (HSV), vaccinia, vesicular stomatitis virus (VSV), measles, maraba, and so on. In addition, every year new candidate viruses are being proposed and investigated for their potential oncolytic abilities (8). Thus far, OVs have been shown to target cancers of almost every possible tissue origin including breast, ovarian, prostate, brain, colorectal, kidney, etc. both *in vitro* and *in vivo*. Considering the capacity of OVs to target cancer cells preferentially, many of these OVs are employed as anti-cancer agents to target various cancers and are currently under phase I, II, and III clinical trials internationally (8–12).

The primary mode of action for OVs is direct oncolysis. In recent years, however, another aspect of OV-based oncotherapy has become evident. Many reports have shown that, in addition to their direct oncolytic activities, OVs aid in the development of tumor-specific T-cell responses (13–20). Thus, if appropriately managed, OV-based oncotherapies can target cancers through two distinct mechanisms: direct oncolysis and anti-tumor immune responses.

The induction of antigen-specific T-cell response begins when antigen presenting cell (APC) presents an antigenic peptide to a naïve T cell. In the absence of a successful antigen presentation event, T cells either remain inactivated or become dysfunctional. Hence, the process of antigen presentation is a critical step during the initiation of T-cell response. Here, we first explain how the components of the APC–T-cell interaction work, then discuss how cancer cells avoid the presentation of tumor antigens, and finally elucidate how the OV-driven immunological events influence the tumor antigen presentation. We believe that the comprehensive understanding on this aspect of OV-based oncotherapy will advocate the development of a clinically meaningful anti-tumor immunity and consequently promote better cancer outcomes.

## Components of the Normal Antigen Presentation Process

**Figure 1:**
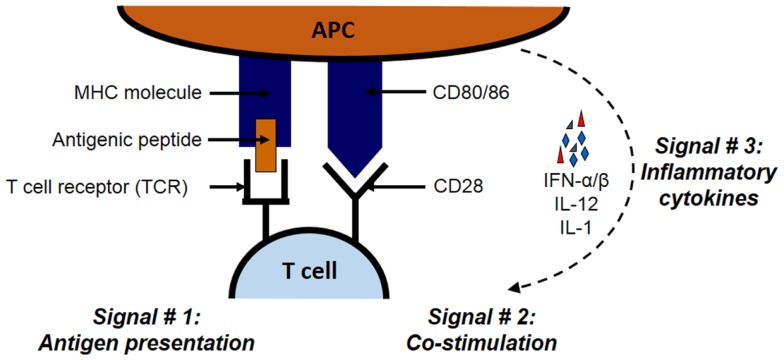
**The three signals necessary for the stimulation of antigen-specific T cell**. The priming of antigen-specific T cell requires three signals: antigen, co-stimulation, and cytokines. Antigenic proteins undergo antigen processing and then the peptides are presented through MHC class I or II molecules for CD8 and CD4 T cells, respectively. The second signal in the form of co-stimulation is provided by molecules such as B7 family member proteins such as B7.1 (CD86) and B7.2 (CD80) expressed on APCs. These B7 proteins interact with their receptors such as CD28 on interacting T cells. Inflammatory cytokines such as IFN-α/β, IL-12, and IL-1 constitute the third signal.

As illustrated in Figure 1, the priming of antigen-specific T cell occurs in lymphoid tissues and requires three signals on APCs: antigen, co-stimulation, and cytokines. Antigenic peptides are presented through major histocombatibility complex (MHC) molecules, co-stimulation is carried out by co-stimulatory molecules such as B7 family member proteins, and cytokines such as interferon (IFN)-α/β, interleukin (IL)-12, and IL-1 constitute the third signal. Both CD8 and CD4 cells bear distinct receptors (called T-cell receptors; TCRs) that interact with MHC class I or II molecules, respectively (22–26). Class I and II MHC molecules have distinct pathways through which proteins are processed and ultimately presented to T cells. For MHC class I pathway, cytosolic proteins go through the antigen processing and presentation machinery (APM), which is made up of peptide transporters, chaperone proteins, and the Golgi complex. First, proteasomes break down designated ubiquitinated proteins into peptides of 2–25 amino acids in length. These peptides are transported with the help of peptide transporters (TAP1/TAP2) into the endoplasmic reticulum (ER), where they are further trimmed to 8–10 amino acid length to fit within the MHC groove (27–30). Next, chaperones such as calnexin, calreticulin, ERp57, and tapasin aid the loading of the trimmed peptide into the MHC groove. These MHC–peptide complexes then migrate to the cell surface and become available for the recognition by CD8 T cells (21, 30).

Apart from this classical pathway, extracellular antigens can also be presented through MHC class I pathway using a specialized pathway called cross-presentation (21, 31). *In vitro*, various APCs have shown to bear a capacity to cross-present extracellular antigens; however, *in vivo*, the main mediators of cross-presentation are DCs (32). There are two main pathways through which cross-presentation can happen: cytosolic and vacuolar. In the cytosolic pathway, first antigen processing occurs in cytosol and then proteasome-generated peptides are fed in MHC class I molecules. On the other hand, for vacuolar pathway, lysosomal proteolysis contributes toward peptide generation, and antigen processing and peptide loading occurs in endocytic compartments. Together, both these pathways facilitate the presentation of extracellular antigens, e.g., antigens from the pathogens that do not infect DCs or self-antigens, to CD8 T cells (33–35).

The expression of MHC class II is more tightly regulated than MHC class I and is primarily found on the surface of professional APCs, such as DCs and macrophages (21). MHC class II antigen processing primarily uses a lysosomal pathway that degrades proteins taken up by endocytosis (extracellular antigens) or autophagy (intracellular antigens). The newly synthesized MHC class II molecules assemble with a protein known as an invariant chain (li). The li protein prevents the premature binding of endogenous peptides or misfolded proteins in the MHC class II groove, and also directs delivery of MHC molecules to endosomal vesicles where the loading of the appropriate peptide happens. Once inside the endosomal vesicle, the li is cleaved off, leaving a short class II-associated invariant chain peptide (CLIP) fragment still bound in the MHC groove. Finally, the release of the CLIP fragment and the loading of the appropriate peptide are facilitated by HLA-DM (H-2M in mouse) molecules (36). The MHC class II molecule displays the appropriate peptide and then travels to the surface to be available for CD4 T-cell recognition (21, 34, 37, 38).

The second signal in the form of co-stimulation is induced when molecules such as B7.1 (CD80) or B7.2 (CD86) expressed on the same MHC–peptide bearing APC interact with its cognate receptor such as CD28 on the interacting T cell (39–42). Other similar co-stimulatory molecule–receptor interactions include the dialogs between CD40L and 4-1BB (CD137) on T cells and CD40 and 4-1BB ligand (4-1BBL) on APCs, respectively. On the other hand, molecules like CTLA-4 on T cells can also bind to B7 molecules and induce inhibitory signals that are especially important in preventing unchecked, sustained proliferation following the initiation of T-cell response. Indeed, mice lacking *CTLA-4* gene display massive proliferation of lymphocytes which becomes fatal overtime (41). Together, the balanced actions of these co-stimulatory and co-inhibitory molecules dictate the fate of T-cell activation.

In recent times, the third signal in the form of inflammatory cytokines has been recognized for the activation of both CD4 and CD8 T cells (43, 44). Cumulative evidence demonstrates that IFN-α/β and IL-12 are required as the third signal for the functional activation of CD8 T cells (43, 45, 46), and that the absence of these cytokines results in the development of defective CD8 T primary and memory responses (47). For CD4 T cells, this third signal is provided by IL-1 (43, 48).

When naïve CD8 or CD4 T cells interact with APCs expressing both the necessary signals, they undergo clonal expansion and differentiate into effector cells. Activated CD8 cells can kill target cells through perforin, granzyme, or FasL-mediated mechanisms or can produce cytotoxic cytokines such as IFN-γ or tumor necrosis factor alpha (TNF-α). On the other hand, activated CD4 cells can also kill target cells or further provide “help” for the activation of other immune cells including macrophages and (T and B) lymphocytes through the action of cytokines such as TNF-α, IFN-γ, granulocyte macrophage colony-stimulating factor (GM-CSF), CD40L, IL-4, IL-5, IL-10, and transforming growth factor beta (TGF-β). Most importantly, a fraction of primed T cells further evolves into a memory phenotype that establishes protection against the same immunogen in the future (23, 26, 49, 50).

## Tumor-Associated Impairment of Antigen Presentation

Tumors have developed various immune evasion mechanisms that specifically target different aspects of signal 1, 2 or 3, and thus prevent the initiation of functional tumor-specific T-cell response (51, 52). More importantly, such defects in antigen presentation and co-stimulation processes, alone or in combination with each other, have been correlated with poor cancer outcomes (17, 30, 37). These defects, which can occur on tumors themselves or on the tumor-associated APCs, have been observed at the transcriptional and/or post-transcriptional levels, and are affected by genetic and environmental factors. For example, completely absent or aberrant expression of MHC class I as well as its constituent protein β2 microglobulin (β2M) has been reported in patients with breast, ovarian, cervical, skin, esophageal, and colorectal cancers (30, 52, 53). Furthermore, other components of the APM such as transporter proteins TAP1 and TAP2, ER enzymes (ERAP1 and ERAP2), proteasome subunits (LMP2, LMP7, and LMP10), and chaperone proteins have been found to be defective in various cancers (4, 5, 30, 51, 54). Unlike MHC class I, the clinical significance of MHC class II expression on tumor cells is still not clear (36). Many tumor cells display constitutive or inducible levels of MHC class II (3, 4, 38). Breast and colorectal carcinomas express MHC class II molecules on the surface; however, they often display the defects in the expression of MHC class II pathway-associated components (55). In contrast to healthy cells, melanoma cells do not upregulate the expression of MHC class II following IFN-γ stimulation. Recently, defects in MHC class II transactivator (CIITA) synthesis was associated with impaired MHC class II expression in head and neck cancer cells and some lymphomas (55–58). Similarly, the impaired levels and functional attributes of HLA-DM and HLA-DO are known to influence the presentation of tumor antigens through MHC class II pathway (36, 55). In the context of such aberrant MHC expression, both CD4 and CD8 cells cannot identify tumors as targets.

Tumor-associated APCs also demonstrate defects in their antigen presentation capacities and could directly contribute toward the establishment of dysfunctional anti-tumor immune response (52). Of note, tumor cells as well as their microenvironment promote an immunosuppressive environment that prohibits the generation of one or more of the three signals of antigen presentation on APCs (52, 54). For example, intra-tumoral DCs obtained from cancer patients or cancer-bearing experimental animals display lower expression of MHC class I and II as well as CD80 and CD86 molecules (51, 52, 54, 59). Similar aberrant expression of MHC and co-stimulatory molecules can be induced on the DCs isolated from healthy, non-cancer-bearing hosts when incubated in the presence of cancer cells and supernatant from cancer cell cultures (17). Additionally, tumor-associated DCs also express various inhibitory molecules, such as programed death ligand-1 (PDL-1) and CTLA-4, which further contribute toward the silencing of anti-tumor T-cell response (41, 42). Finally, tumor microenvironment also recruits many suppressive cells [e.g., regulatory T cells (Tregs) and myeloid-derived suppressor cells (MDSCs)] and cytokines (e.g., TGF-β, PGE-2) which further affect the antigen presentation function of APCs (51, 52).

## Contribution of Virus-Driven Immune Response in the Antigen Presentation Process

Viruses are strong immunogens, and bear a capacity to induce all three signals, i.e., antigen, co-stimulation and inflammatory cytokines, necessary for the activation of antigen-specific T-cell response (60). Following exposure to a virus, the immune system recognize the virus as a “foreign” entity through conserved receptors of the innate immune system known as pattern recognition receptors (PRRs, e.g., toll-like receptors, TLRs). These receptors on APCs can identify molecular motifs known as pathogen-associated molecular patterns (PAMPs) and virus-associated DNA and single- or double-stranded RNA of genomic or replicative intermediate origin. Additionally, replicating viruses are also recognized through intracellular helicases (60, 61). The recognition of viral PAMPs through PRRs drives the immediate innate immune response that constitutes the production of type I interferons, including multiple forms of IFN-α and -β (62–64). These Type I interferons enhance the expression of MHC class I and II, CD40, CD80, CD83, and CD86 on the surface of DCs (46, 65, 66). Such IFN-α/β response further stimulates the production of cytokines (e.g., IL-1β, IL-6, IL-12, TNF-α) and chemokines [e.g., IL-8, monocyte chemotactic protein-1 (MCP-1)], and amplifies the initial innate response when these cytokines act through autocrine and paracrine fashion (67). This cytokine-driven pro-inflammatory response is critical in driving the expression of MHC as well as co-stimulatory molecules involved in antigen presentation. Of note, IFN-α has been shown to enhance the proliferative capacity of naïve CD8 T cells, and thus is considered as a “signal 3” necessary for successful T-cell activation (44). Additionally, this innate response is also known to promote the cross-presentation of antigens (3, 68). The APCs primed in this fashion travel to the lymphoid organs wherein they interact with naïve T cells and prime an antigen-specific adaptive immune response (34).

## OV-Mediated Reversal of Tumor-Associated Impaired Antigen Presentation

The immune responses that accompany oncolytic virotherapy warrant a special consideration as the circumstances under which these responses occur are very unique to this system. It should always be remembered that OV-driven immune responses are strong, whereas cancers usually persist in suppressive environments. The combination of these two contrasting entities most likely produces the immunological consequences that are uncharacteristic of either the tumor- or virus-driven immune response on their own (14). Interestingly, OVs preferentially target cancer cells for their replication, and hence attract the anti-viral immune response in a cancer microenvironment (14, 69, 70).

**Figure 2:**
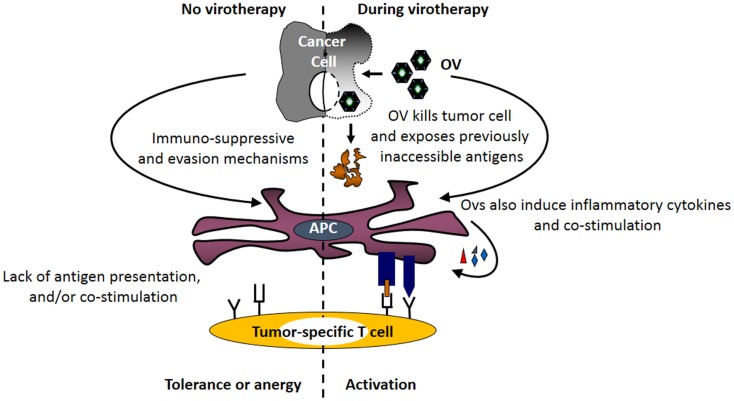
**Oncolytic viruses facilitate the tumor antigen presentation preceding the initiation of anti-tumor immunity**. Following its therapeutic administration, OVs enhance the expression of MHC molecules on tumor and immune cells. At the same time, OV-mediated direct oncolysis of tumor cells exposes tumor-associated antigens (TAAs) for the processing by APCs. Through the combined actions of these immunological events, OVs facilitate the display of otherwise inaccessible tumor-specific immunogenic peptides on the surface of APCs (*generation of signal no. 1*). Additionally, OV-induced inflammatory response promotes the expression of co-stimulatory molecules on APCs (*generation of signal no. 2*) and production of inflammatory cytokines (*generation of signal no. 3*). Together, OV-driven immunological events over-turn tumor-associated antigen presentation impairments, and initiate anti-tumor immunity.

The strong immune responses initiated by viruses have the potential to over-turn the suppressive effects of tumor-associated immune evasion mechanisms (Figure 2), including those involved in antigen processing and presentation pathway (71–74). Exposure of immune as well as cancer cells to OVs induces the expression of type I interferons (75). Similarly, animals injected with the OV gain elevated IFN-α mRNA and protein levels immediately following the administration of the virus. Furthermore, DCs cultured in the presence of reovirus produce IL-1α, IL-1β, IL-6, IL-12p40/70, IL-17, CD30L, eotaxin, GM-CSF, MCP-1, MCP-2, MCP-5, macrophage colony-stimulating factor (M-CSF), monokine induced by gamma interferon (MIG), macrophage inflammatory protein-1 alpha (MIP-1α), RANTES, TNF-α, vascular cell adhesion protein-1 (VCAM-1), etc., and show enhanced expression of CD80, CD86, and CD40 (71). Similar phenotype is also observed in DCs exposed to other OVs including HSV, vaccinia, and measles (72, 76–78). Most importantly, APCs isolated from the spleens of the tumor-bearing mice injected with a therapeutic regimen of OVs also display higher expression of co-stimulatory molecules as compared with those isolated from the untreated or PBS-injected tumor-bearing animals (71, 79). It should be noted that DCs isolated from tumor-bearing mice have lower expression of co-stimulatory molecules as compared with their healthy counterparts. However, this lowered expression is over-turned following OV administration (17, 71).

Most OVs are potent inducers of MHC class I pathway-related molecules (13, 14, 18, 19, 80). Exposure of tumor cells to OVs *in vitro* enhances the expression of MHC class I molecules as compared with that observed in untreated cells (17). For example, when mouse ovarian tumor cells (ID8), which show complete absence of MHC class I protein on its surface under native conditions, manifest significantly higher MHC class I expression upon exposure to reovirus for 24 h in vitro (17). Furthermore, ID8 tumors collected from reovirus-treated C57BL/6 immuno-competent mice also displayed significantly higher expression of mRNA transcripts encoding MHC class I, β2M and TAP1/TAP2, molecules as compared with that of tumors from untreated animals (17).

From a functional point of view, OVs are known to directly enhance the antigen presentation capacity of DCs (71). When DCs are incubated in the presence of OV-infected ova-expressing tumor cells, they can efficiently process and present a tumor-associated antigen (TAA) to antigen-specific CD8 T cells. This was shown in a cancer model wherein an ovalbumin (ova) is employed as a surrogate tumor antigen. In this model, when bone marrow-derived dendritic cells (BMDCs) are incubated with reovirus-infected ova-expressing mouse melanoma (B16-ova) or lung carcinoma (Lewis lung carcinoma, LLC-ova) cells, they display the ova-specific immune-dominant epitope in the context of MHC class I molecules on their surface. Such display of surrogate TAA is non-existent when BMDCs are incubated with B16-ova or LLC-ova in the presence of inactivated virus or medium alone. Most importantly, OV-induced TAA presentation on the BMDC surface further stimulates the activation of TAA-specific CD8 T cells (71). These observations conclusively demonstrate that OVs can (1) promote the antigen presentation of TAAs on APCs and (2) endow APCs with a functional capacity to stimulate TAA-specific CD8 T cells. Of note, the use of ova as a surrogate TAA should be cautiously considered as it could potentially undergo differential antigen processing and presentation than that for endogenous TAA.

The over-turning of the tumor-associated impaired antigen presentation, however, is only observed following exposure to live, but not inactivated, OVs (71, 72, 81), and is thought to be directly associated with the process of oncolysis. It is believed that OVs expose otherwise inaccessible tumor antigens through oncolysis and make them available to APCs. Simultaneously, OV-driven inflammatory response also promotes the expression of co-stimulatory signals on these APCs that are now armed with tumor antigen. Thus, oncolytic activities of OV coupled with virus-driven immunological events induce the signals necessary for the activation of tumor-specific T cells and aid in the development of anti-tumor adaptive immunity.

Nevertheless, not all OVs aid in the antigen presentation process. Thus far, VSV has been shown to downregulate the co-stimulatory and antigen presentation functions, along with the survival of DCs (82). This observation bears special significance especially in the context of the capacity of various other viruses to subvert and manipulate antigen presentation pathways (53, 68, 83, 84). Hence, it is imperative that candidate OVs be tested extensively for their respective beneficial or detrimental immunological capacities related to the process of tumor antigen presentation.

## Future Directions

As outlined in this perspective, OVs bear a comprehensive capacity to over-turn TAA presentation evasion mechanisms and to promote a functional anti-tumor T-cell response. However, available information on this phenomenon is still limited and warrants a detailed exploration on various molecular and functional aspects of OV-driven antigen presentation. Especially, the effect of OVs on the processing and presentation of endogenous tumor antigens in the context of various molecular components of MHC class I and II pathway, and in relation with resultant anti-tumor immune response, must be thoroughly explored. It should also be noted that OV-induced antigen presentation also promotes the development of the anti-viral adaptive immune response that is known to prematurely curtail the spread of OV in cancer cells. Only in recent years, the importance of OV-driven immunological events has been acknowledged and given appropriate attention. However, one thing is now clear: OV-induced immune response dictates the efficacy of OV-based oncotherapy. In the future, appropriate immune interventions that promote a fine balance between anti-tumor and anti-viral immune responses will ensure the maximum anti-cancer benefits of OV-based oncotherapies.

## Conflict of Interest Statement

The authors declare that the research was conducted in the absence of any commercial or financial relationships that could be construed as a potential conflict of interest.
